# Merging Worlds of Nanomaterials and Biological Environment: Factors Governing Protein Corona Formation on Nanoparticles and Its Biological Consequences

**DOI:** 10.1186/s11671-015-0922-3

**Published:** 2015-05-16

**Authors:** Parisa Foroozandeh, Azlan Abdul Aziz

**Affiliations:** School of Physics, Universiti Sains Malaysia, 11800 Penang, Malaysia; Nano-Biotechnology Research and Innovation (NanoBRI), Institute for Research in Molecular Medicine (INFORMM), Universiti Sains Malaysia, 11800 Penang, Malaysia

**Keywords:** Protein corona, Nanoparticle, Bio-nano interface, Ignored factors

## Abstract

Protein corona has became a prevalent subject in the field of nanomedicine owing to its diverse role in determining the efficiency, efficacy, and the ultimate biological fate of the nanomaterials used as a tool to treat and diagnose various diseases. For instance, protein corona formation on the surface of nanoparticles can modify its physicochemical properties and interfere with its intended functionalities in the biological microenvironments. As such, much emphasis should be placed in understanding these complex phenomena that occur at the bio-nano interface. The main aim of this review is to present different factors that are influencing protein-nanoparticle interaction such as physicochemical properties of nanoparticle (*i.e.*, size and size distribution, shape, composition, surface chemistry, and coatings) and the effect of biological microenvironments. Apart from that, the effect of ignored factors at the bio-nano interface such as temperature, plasma concentration, plasma gradient effect, administration route, and cell observer were also addressed.

## Review

### Introduction

Nanoscience is recognized as a promising field of science that can overcome several scientific shortcomings in diverse scientific fields, such as physics, biology, chemistry, and materials science [1, 2]. The breakthroughs achieved in the field of nanoscience are mainly attributable to the changes in the properties of materials as they are reduced to the size of nanometer from their bulk form. The materials assume novel mechanical, chemical, electrical, optical, magnetic, electro-optical, and magneto-optical properties as compared to bulkier counterparts [3–5]. For instance, gold in bulk form is inert and conducts electricity, however gold shrunk into “nano” form becomes a very good catalyst and turns into a semiconductor instead. One of the most exciting prospects that have emerged from the field of nanoscience is nanoparticle (NP) technology that are currently being incorporated and utilized to solve many intricate technical problems in modern science, chiefly in the field of medicine and biomedical science, which has given birth to the term nanomedicine. Application of NP in medical biology arises from their ability to encounter cellular machinery and potentially access to unreachable targets like the brain due to their small size [6, 7]. Consequently, they have shown promising application in various branches of biomedical science such as drug delivery [8], gene delivery [9–11], tissue repair [12], cancer therapy, [13] disease diagnoses and therapy [14], hyperthermia [15], magnetic resonance spectroscopy [16], and as contrast agents for magnetic resonance imaging (MRI) [17].

NPs owing to their large surface-to-volume ratio have a very active surface chemistry in comparison to bulk materials. When they come into contact with biological milieu, they seek to lower their high surface energy by adsorbing biomolecules, resulting in the creation of complex layer of biomolecules that would cover the surface of NP [18–20]. More specifically, when NPs are exposed to a biological medium, physical and chemical interactions occur between the surfaces of NPs and different biological components within the medium such as proteins, peptides, and glycolipids. Due to these interactions, a “bio-nano interface” develops at the point where the two entities come into contact [21–23]. The formed interface covers the surface of NP, thereby modifying its quality and endowing it an identity within a biological framework, the so-called protein corona. Interestingly, the biological identity dictates the cellular/tissue responses such as cellular uptake, kinetics, signalling, accumulation, transport, and toxicity [19, 22, 24, 25]. Also, the protein corona gives information about the interface formed between the NP and the biological milieu [25]. Walkey and his fellow researchers employed protein corona fingerprint to establish a quantitative model which will predict the cell association of various composition of gold NPs [26]. Their findings suggest that this model is 50 % more accurate compared to the one which applies NP parameters such as size, aggregation state, and surface charge indicating that the protein corona gives more information about the biological behavior of NP rather than its physical properties.

Protein corona can exist in two different forms on the surface of NPs, determined by the type of layers formed. Essentially, two different type of layers can be formed, namely “soft” and “hard” coronas, the former consisting of loosely bound proteins with short lifetime and the latter consisting of tightly bound proteins with long lifetime [27]. Composition of protein corona is influenced by the physicochemical properties (*i.e.*, composition, size, shape, and surface properties) of NPs and the characteristic of biological environment in which the NPs are dispersed. It is noteworthy to mention that the cell perceives protein corona as opposed to the bare surface of NPs as they come into contact [21, 24, 28]. Thus, understanding protein corona which confers the biological identity to NP is important as it will have major repercussions in the field of nanomedicines whereby toxicological and physiological responses to NPs are studied.

This review presents different factors that are influencing protein-NP interactions, including the effect of physicochemical properties of NP (*i.e.*, size and size distribution, shape, composition, surface chemistry, and coatings), effect of environment, and the effect of ignored factors at the bio-nano interface such as temperature, plasma concentration, plasma gradient effect, administration route, and cell observer. Moreover, the impact of these parameters on the composition of protein corona and fate of NPs in biological environment will be addressed.

### Creation of Protein Corona

It is now well understood that upon coming into contact with NPs in the biological medium, different biomolecules such as proteins, lipids, and glycans will compete to interact with the NP surface to form a layer called protein corona [23, 24, 29]. For instance, in the case of intravenous administration of the NPs, blood plasma proteins adsorb to the surface of NP to form protein corona [23, 30–32]. In the case of other administrative routes, NPs will react with other biomolecules of the body fluids primarily before reaching the blood plasma. Protein corona is a dynamic layer and its composition changes with time due to ongoing protein absorption and desorption [19, 29]. It is worth remarking that protein corona is the primary contact to the cell. Therefore, what the biological entity sees when it comes into contact with NPs is the protein corona formed at that specific time [19, 29, 33]. The composition of protein corona for each nanomaterial is unique and is influenced by many parameters such as physicochemical properties of NPs and characteristics of the environment [25].

The formation of protein corona causes a reduction in the surface energy and toxicity of the NP as compared to the “bare” particle. In general, protein corona changes the size, surface chemistry, and surface charge of the NP, thereby affecting its uptake, biodistribution, and cellular fate [18, 24]. Protein corona is formed at the bio-nano interface by the aid of several forces such as hydrodynamic, electrodynamic, and electrostatic or steric forces and solvent and polymer bridging. These forces also determine the kind of structure that protein corona may form at these interfaces. Structure of protein corona can be evaluated in the physiological environment (*i.e.*, in situ) or after isolation from the physiological environment (*i.e.*, ex situ). Techniques such as differential centrifugation (DC) or size exclusion chromatography (SEC) are utilized for isolation of protein corona. Different parameters of protein corona such as thickness, protein identity, protein quantity density, protein-NP affinity, and protein conformation can be analyzed and quantified using various analytical tools [24, 25].

### Composition and Structure of Protein Corona

The protein corona evolves over time from what was formed at the initial stage of NPs’ interaction with biological medium. Initially, when the NPs come into contact with biological medium, the most abundant proteins with low affinity adsorb to the NP surface and form the layer which is called soft corona. Over time, those proteins would be replaced by high affinity proteins that have a lower abundance in the medium and form the hard corona layer [22, 30, 34, 35].

**Fig. 1:**
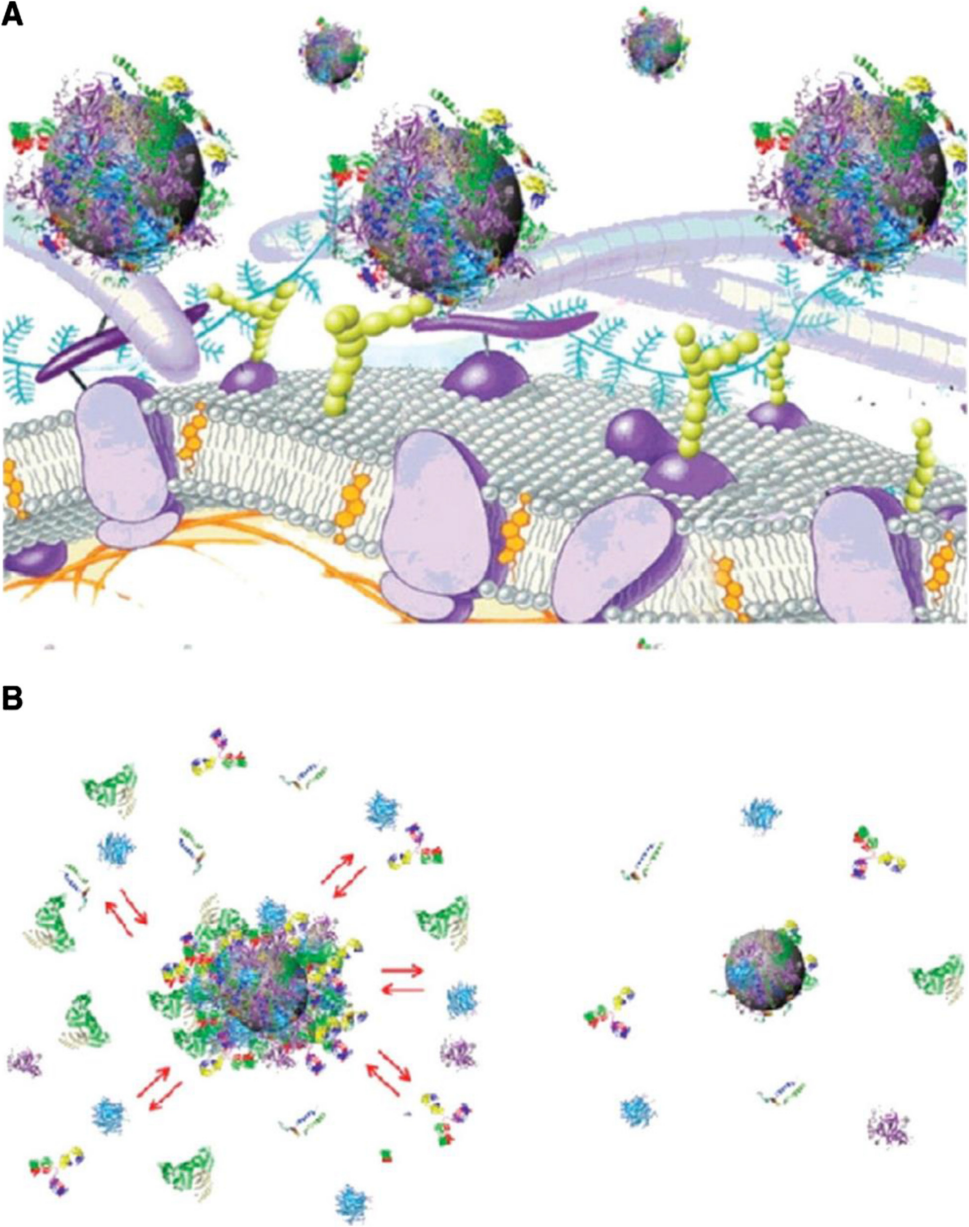
Schematic representation of exchange/interaction scenarios and of the structure of protein-nanoparticle. **a** Schematic representation of the possible exchange/interaction scenarios at the bionanointerface at the cellular level. **b** Schematic drawing of the structure of protein-nanoparticle in blood plasma confirming the existence of various protein binding (*e.g.*, an outer weakly interacting layer of protein (*full red arrows*) and a hard slowly exchanging corona of proteins (*right*) (adapted with permission from [35]))

The coronas may also be classified based on their exchange rates. In particular, hard corona possess long lifetime which shows slow exchange rate with the medium while soft corona has faster exchange rates. Hard corona consists of tightly bound proteins that do not easily desorb, in contrast to weakly bound proteins that constitute the soft corona. It is well accepted that owing to the longer half-life of hard corona in the biological medium, its interaction with cellular receptors will determine the fate of NPs as depicted in Fig. 1 [20, 25, 36, 37].

It is now hypothesized that either the proteins in the hard corona adsorb directly to NPs surface while proteins in the soft corona bind to the hard corona via weak protein–protein interaction or the hard and soft corona proteins may both bind directly to the NPs surface with distinct binding energies [25]. Hard corona does not exist for all nanoparticles. Predominantly for NPs that are coated with functional group such as PEGylated nanoparticles, only the soft corona can be observed [38]. Due to long residence time of proteins in the hard corona, it is considered to be the main component in defining the biological identity for NPs.

Simberg and co-workers introduced a model for the protein corona which includes “primary binders” that interact with the NPs surface at first followed by “secondary binders” that binds to the primary binders by way of protein–protein interactions [39]. This multilayered structure plays an important role in the physiological response as the interaction of the primary binders can be changed by the secondary binders or being “masked” by them, thereby hindering their interaction with the biological environment.

Walky and Chan summarized the composition of the protein corona across 26 studies for 63 nanomaterials, and they have identified a subset of 125 unique plasma proteins that have adsorbed to at least one nanomaterial [25]. This subset of plasma proteins was identified and categorized as “adsorbome.” One could observe that each “adsorbome” have different physiological roles, but they commonly participate in lipid transport, complement activation, pathogen recognition, blood coagulation, and ion transport. The authors have established that at high abundance roughly 2–6 proteins are adsorbed in a “typical” plasma protein corona and at low abundance, more protein are absorbed. A review study summarized the types of protein binding to different nanoparticles to determine the composition of protein corona [40]. This study postulates that albumin, immunoglobulin G (IgG), fibrinogen, and apolipoproteins can be detected in the corona of all the nanoparticles due to their high abundance in plasma.

Variation in composition of protein corona over time is explained by the Vroman effect. The Vroman effect states that the composition of protein corona may vary over time whereas the total amount of protein remains relatively constant. In particular, the Vroman effect elucidates how low affinity but highly abundant proteins that are adsorb first to the surface of the NP will be replaced by higher affinity proteins. The Vroman effect is the function of concentration of protein, incubation time, and affinity of proteins. Upon intravenous administration of NPs, the Vroman effect in plasma involves the adsorption of high abundance proteins such as albumin, IgG, and fibrinogen which is called “early” stage. The “late” stage of the Vroman effect occurs when those proteins will be replaced by high affinity proteins such as apolipoproteins and coagulation factors [41–44].

Goppert and co-workers investigated “Vroman effect” on solid lipid nanoparticle (SLN) over a period of time (*i.e.*, 0.5 min to 4 h) [45]. They reported that at the early stages, albumin was replaced by fibrinogen. The longer incubation time resulted in the replacement of fibrinogen with IHRP (inter-α-trypsin inhibitor family heavy chain-related protein) and apolipoproteins. Their study have demonstrated that protein desorption did not occur from SLN throughout the time period of their investigation on the protein adsorption kinetics. They also observed an increase in the total amount of proteins that were adsorbed onto the surface of SLN after more than 4 h of incubation with plasma. The on oil-in-water nanoemulsions (o/w nanoemulsions) showed a markedly different adsorption behavior as compared to SLN whereby the originally adsorbed proteins were replaced by proteins having a higher affinity to the surface (“Vroman effect”). No Vroman effect could be observed on oil-in-water nanoemulsions (o/w nanoemulsions). Furthermore, increasing plasma concentration leads to an increment in the amount of adsorbed apolipoproteins A-I, A-IV, C-II, and C-III [42]. The Vroman effect on ultrasmall superparamagnetic iron oxide (USPIO) nanoparticles has been assessed by Jansch and co-workers [46]. Their study showed that no Vroman effect on USPIO can be determined and no replacement of higher affinity protein with high abundance protein on USPIO could be detected. Moreover, with prolonging incubation time, the amount of fibrinogen and immunoglobulins increased while the relative amount of major proteins, such as apolipoproteins, fibrinogen, and albumin, remained constant over time.

### Protein Corona Conformation

When proteins adsorb to NP, structural rearrangements may take place within the protein molecules. These “conformational changes” can render the protein to become dysfunctional due to loss of native form or thermodynamically favorable if it allows either charged or hydrophobic regions of proteins to interact with either hydrophobic or charged NPs [25, 47, 48]. It is remarkable to note that conformational changes of proteins after desorption are usually irreversible. However, the structure of protein and the surface properties of NPs are key factors in determining the level of conformational change. For instance, hydrophobic nanoparticles undergo the conformational change more than hydrophilic NPs [49]. Mahmoudi and co-workers illustrated the conformational changes of iron-saturated human transferrin protein due to interaction with superparamagnetic iron oxide NPs (SPIONs) [50]. Different sizes of bare SPIONs and PVA-coated SPIONs were incubated with iron-saturated human transferrin protein. Analyzing the resulting complex by fluorescence and UV-vis spectroscopy, SDS-PAGE gel electrophoresis and circular dichroism revealed irreversible changes in conformation of iron-saturated human transferrin protein due to the release of iron. Particularly, conformation of human transferrin changes from a compact structure to an open structure. Moreover, it was found that conformational changes of transferrin depend on the surface properties and size of the SPIONs. For instance, more conformational changes were detected on the bare NPs than PVA-coated NPs.

Shang and co-workers studied the conformational changes of bovine serum albumin (BSA) in the albumin to gold nanoparticle bioconjugates at different pH values (*i.e.*, 2.7 (E form), 3.8 (F form), 7.0 (N form), and 9.0 (B form) by employing different spectroscopic techniques such as UV-vis, fluorescence, circular dichroism, and Fourier transform infrared spectroscopies [51]. The results show that the changes in the conformation of BSA occur at the secondary and the tertiary structure levels. In addition to that, studies on the effect of environmental pH on the conformational changes indicated that higher pH causes larger changes.

### Time Evolution of the Protein Corona

The time evolution of the protein corona formed on different-sized gold NPs ranging from 4 to 40 nm in the cell culture media with 10 % fetal bovine serum (FBS) was demonstrated by Casals and co-workers [52]. Protein corona formed around gold NPs were analyzed by zeta potential measurements, UV-vis spectroscopy, dynamic light scattering, and transmission electron microscopy which revealed a reduction of surface charge and an increment in the thickness of protein corona layer. Albumin was shown to be the most abundant protein on the surface of NPs by mass spectrometry analysis of the protein corona. Furthermore, they reported that loosely bound proteins will over time evolve to form an irreversible bound protein. The evolution of protein corona after NPs were transferred from plasma into cytosolic fluid was illustrated by Lundqvist and co-workers [38]. Various NPs such as 9-nm silica, 50-nm polystyrene, and 50-nm carboxyl-modified polystyrene particles were employed in this study. It was observed that the protein corona considerably evolves in the second biological medium. Yet, the final corona preserves a “fingerprint” of prior history. This can be beneficial to determine the transport pathways of NPs.

The changes in the adsorption pattern of serum proteins over time from 5 to 360 min were quantitatively and qualitatively investigated by Nagayama and co-workers [53]. They used SDS-PAGE and Western blotting to asses 50-nm lecithin-coated polystyrene NP at different times. The quantitative study revealed that the total amount of adsorbed proteins increased over time. The qualitative study indicated variation in the kinds of proteins adsorbed since the amount of some proteins increased over time whereas others decreased. Complement C3, IgG, apolipoprotein E (ApoE), and immunoglobulin A (IgA) showed increment over time. On the contrary, concentration of albumin remained constant.

### Effect of Different Parameters on the Composition of Protein Corona

Several factors affect the manner by which NPs interact with biomolecules and composition of the resulting protein corona. In view of the fact that protein adsorption takes place at the interfacial region between NPs and its surroundings, the physicochemical properties of NPs and the biological environment are vital parameters governing protein corona formation. Therefore, analyzing and understanding each of these parameters are essential for safe design of NPs.

#### Effect of Nanoparticle Composition

NP composition and its surface chemistry are crucial factors in determining the affinities and identities of proteins that bind to NPs. Deng and co-workers have studied the binding of human plasma proteins to commercially available metal oxide NPs such as titanium dioxide, silicon dioxide, and zinc oxide with the same surface charge [54]. The authors revealed that similar proteins adsorb to titanium and silicon dioxide NPs, whereas significantly different proteins composed the hard corona of zinc oxide NPs. In particular, clusterin, apolipoprotein D, and alpha-2-acid glycoprotein were detected in the corona of titanium and silicon dioxide NPs while those were not observed in the corona of zinc oxide. Interestingly, some other proteins like transferrin, Ig heavy chain alpha, and haptoglobin (alpha) only were found in the corona of zinc oxide NPs alone.

#### Effect of Nanoparticle Size

The surface curvature of NP, which is associated to the size of the NP, plays a key role on the adsorption of proteins, conformational changes and composition of protein corona [32, 55]. Due to high surface curvature of NP as compared to the bulk materials, their protein binding affinities are distinct from bulk materials of the same composition [56]. More specifically, protein–protein interactions reduce at high curved surfaces of nanoparticle leading to different composition of protein corona. In addition, adsorbed proteins at highly curved surface NPs undergo less conformational changes than adsorbed proteins at flat surfaces of the same material [19, 24]. Tenzer and co-workers investigated the protein corona formation on silica NPs (SiNP) of various sizes upon exposure to blood plasma [57]. They observed that the size of SiNP drastically affected the binding of 37 % of the identified proteins. Interestingly, even 10-nm variations in particle size notably influence protein corona composition. Likewise, lipoprotein clustsacaerin bound to the small SiNP while prothrombin or the actin regulatory protein gelsolin were absorbed on the larger SiNP. However, no correlation to NP size was observed for the adsorption of several proteins such as immunoglobulin (IgG) or actin. Dobrovolskaia and co-workers examined 30- and 50-nm colloidal gold incubated in human plasma, and they found more proteins were adsorbed on the 30 nm than on the 50 nm gold NPs [58].

In a recent work, the effect of NP size on binding constant and hill constant (*i.e.*, the degree of cooperatively of protein-NP binding) was probed [31]. The authors used gold NPs of various sizes ranging from 5 to 100 nm and incubating them with common human blood proteins: albumin, fibrinogen, γ-globulin, histone, and insulin. It was observed that the size of gold NPs significantly correlate to binding constant, *K*, as well as the degree of cooperatively of particle-protein binding (Hill constant, *n*), In particular, the binding constant increases with NP size, whereas the Hill constant seeks to decrease with NP size. Additionally, they have also realized that the thickness of protein corona progressively increases as the size of NP increases. Moreover, conformational change upon association with the NPs showed enhancement with the size of NP.

In an analysis of protein corona formed on 50- and 100-nm polystyrene NPs upon exposure to human plasma, Lundqvist and co-workers showed that the size of NP affect the type of adsorbed proteins to the surface of NP to form corona and subsequently composition of the protein corona [32]. For instance, apolipoprotein B-100 did not adsorb to 50-nm NPs but binds to 100-nm NPs. The impact of the size of silica NPs on the adsorption of lysozyme was studied by Vertegel and co-workers [59]. They concluded that adsorption of lysozyme on 100-nm NP causes more protein unfolding than on 4-nm NP. In particular, the size of the NPs was found to be a crucial factor in determining the structure and function of lysozyme upon adsorption onto silica NPs.

#### Effect of Nanoparticle Shape

The manner by which proteins adsorb onto the surface of NP as well as the biological responses to NP is strongly influenced by the shape of NP. For instance, the shape of gold NPs has a huge effect on their interactions with cell layers; in particular, spherical gold NPs has higher association in cell as compared to rod-shaped gold NPs [60]. The effect of shape of titanium dioxide NPs on protein binding was investigated by Deng and co-workers [54]. The authors found that clusterin and apolipoprotein D were only observed on spherical NPs and were not detected on nanorods or nanotubes.

#### Effect of Surface Functional Group and Coating

In order to prevent absorption of proteins and to control the protein corona composition, the surface of NP can be functionalized with different groups. This confers a “stealth character” to the surface of NP, hence eluding from being observed by immune cells. Appropriate polymers such as polyethylene glycol (PEG) can also be applied to coat the surface of NPs to decrease protein binding and prevent them from being recognized by the RES, the so-called PEGylation. Density of PEG on the surface of NPs can be controlled in order to prolong circulation time in blood. “Siliconate” has also been used to coat the surface of NPs and hinder protein adsorption [61, 62]. Polystyrene NPs with different functional groups (*i.e.*, PEG, amidine, carboxyl, amine, lysine, methyl, and cysteine) were used in cultured endothelium cells [41]. It was concluded that the protein binding capacity to these functionalized surface of NPs demonstrate their tendency to interact with the cells. Additionally, NP-cell association is not influenced by the identity of bound proteins.

Studies on poloxamine 908 coating polystyrene nanospheres revealed a reduction of fibronectin adsorption compared to uncoated nanospheres [63]. Coating of both single-walled carbon nanotubes and amorphous silica particles with Pluronic F127 resulted in the enhancement of dispersion of the NPs but notably decreasing adsorption of serum proteins [64]. Aggarwal *et al.* has summarized the effects of various coatings such as PEG, poloxamer, poloxamine, dextran, Pluronic F127, polysorbate, and poly(oxyethylene) on the protein binding and biodistribution of NPs [40].

#### Effect of Surface Charge

Surface charge of the NP is a crucial factor in determining the protein corona composition and consequentially its eventual fate in the biological system. Positively charged NPs are easily recognized by opsonins resulting in the elimination of these particles by the reticuloendothelial system (RES) and its eventual concentration in the liver and spleen [40, 35]. In order to prevent opsonization, NPs surface can be coated with negatively charged groups leading to a negative zeta potential in the range of 30–50 mV in physiological conditions. When the surface-coated NPs are exposed to biological medium, the adsorbed proteins on their surface cause a large decrease of their zeta potential to 5–10 mV negative [41]. Therefore, the colloidal stability of those complexes is directly related to the nature of the protein corona. A study on gold NPs with positive, negative, and neutral ligands show that in the case of charged ligands (both positive and negative), protein denaturation occurs while the neutral ligands retain the structure of proteins [55].

Gessner and co-workers studied the impact of surface charge density of negatively charged polymeric NPs and found enhancement in plasma protein absorption with an increase in the surface charge density of NPs [65]. Studies on polystyrene NPs demonstrated that proteins with isoelectric points (PI) of less than 5.5 like albumin adsorbed on positively charged particles whereas proteins with isoelectric points of higher than 5.5 like IgG bound to negatively charged particles.

#### Effect of Hydrophilicity/Hydrophobicity

More proteins can adsorb onto the surface of hydrophobic NPs than their hydrophilic counterparts. Moreover, due to high affinity of proteins to hydrophobic NPs rather than hydrophilic NPs, many more adsorbed proteins undergo protein denaturation on the surface of hydrophobic NPs and lose their native structure [66]. Likewise, the binding of apolipoproteins were found to be a major part of the formation of protein corona on hydrophobic NPs whereas hydrophilic NPs typically adsorb IgG, fibrinogen, and albumin [30, 47].

Cedervall and co-workers employed ITC to study the affinity and stoichiometry of protein binding [34]. The authors revealed that as the hydrophobicity of particles increase, it promotes the stoichiometry of proteins. They found that albumin on hydrophobic particles has shorter residence time than hydrophilic ones. Furthermore, surface of hydrophobic particles has higher coverage at equilibrium point.

#### Effect of Biological Environment

In addition to the characteristics of NP, composition of the biological medium in which they interact is a critical factor in determining the composition of protein corona [22]. The impact of media composition on the formation of protein corona was studied by Maiorano and co-workers [67]. They incubated various sized citrate-capped gold NPs with cellular media such as Dulbecco Modified Eagle’s Medium (DMEM) and Roswell Park Memorial Institute medium (RPMI) that were supplemented with the fetal bovine serum (FBS). These are the commonly used cell culture media and they differ in glucose, salt composition, and amino acids. A number of techniques (dynamic light scattering, UV-visible, and plasmon resonance light scattering) were used to evaluate the corona formation on gold NPs mediated by DMEM and RPMI. It was concluded that formation of protein corona by utilizing DMEM is significantly time dependent, while using RPMI leads to distinct dynamics and reduction of protein corona. Protein-NP complexes were also characterized by sodium dodecyl sulfate polyacrylamide gel electrophoresis (SDS-PAGE) and mass spectroscopy, and it was found that protein corona composition does not relate to the amount of serum proteins. Viability assays in both cultured media DMEM and RPMI were performed on two cell lines HeLa (human epithelial cervical cancer cell line) and U937 (human leukemic monocyte lymphoma cell line) for 15-nm gold NPs. Interestingly, significant differences in dynamics, cellular uptake, and biodistribution of protein-NP complexes were observed. More specifically, internalization of protein-NP complexes in cells that were formed in RPMI media was notably higher than those formed in DMEM, resulting in higher cytotoxic effects. Moreover, the protein corona formed in DMEM was more abundant and stable compared to protein corona formed in RPMI. These differences in the protein-NP complexes mediated by different environments would affect the cellular response. Therefore, apart from NP characterization, assessment of cell culture media must be made in order to understand its subsequent interaction with NPs.

The conditioning of cell culture medium and its effect on the biological identity of NPs was investigated by Albanese and co-workers [68]. Complete growth medium that are used for culturing cells *in vitro* are commonly supplemented with serum that contains varying amount of proteins or peptides that have the propensity to form corona on the surface of NPs, thereby altering their physicochemical properties and interaction with the cells. However, as the cells are cultured or incubated with the complete growth medium, the composition of these protein or peptides along with other components may change over time due to cellular metabolic activity of the cells, hence affecting the corona formation on the NPs. Conditioning of cell culture medium refers to exposure of the NPs in an environment that the cells have been cultured for a certain amount of time, thereby containing all the by-products of cellular metabolic activity. This mimics the dynamics of the *in vitro* cellular environment that NPs are exposed to more accurately. From the study, it was shown that cell conditioning causes gold NP aggregation which in turn varies the composition of protein corona that relies on NP size, surface chemistry, and cell phenotype. Moreover, it was demonstrated that dynamic extracellular environment may alter the initial biological identity and consequently the cell uptake.

#### Effect of Ignored Factors

Besides the effect of NP characteristics and biological environment, there are several other influential hidden factors at the bio-nano interface which are significantly effective in determining the composition of protein corona and their subsequent cellular responses. Recently, research has been focused to asses these parameters which are generally called “ignored factors,” and it includes gradient plasma, plasma concentration, cell observer, temperature and cell membrane composition. Detailed research must be carried out to understand these ignored factors as to enable the development of better and effective nanomedicine while preventing unanticipated consequences due to poor formulation of nano-based drugs.

##### Gradient Plasma Effect and Nanoparticle Administrative Route

**Fig. 2:**
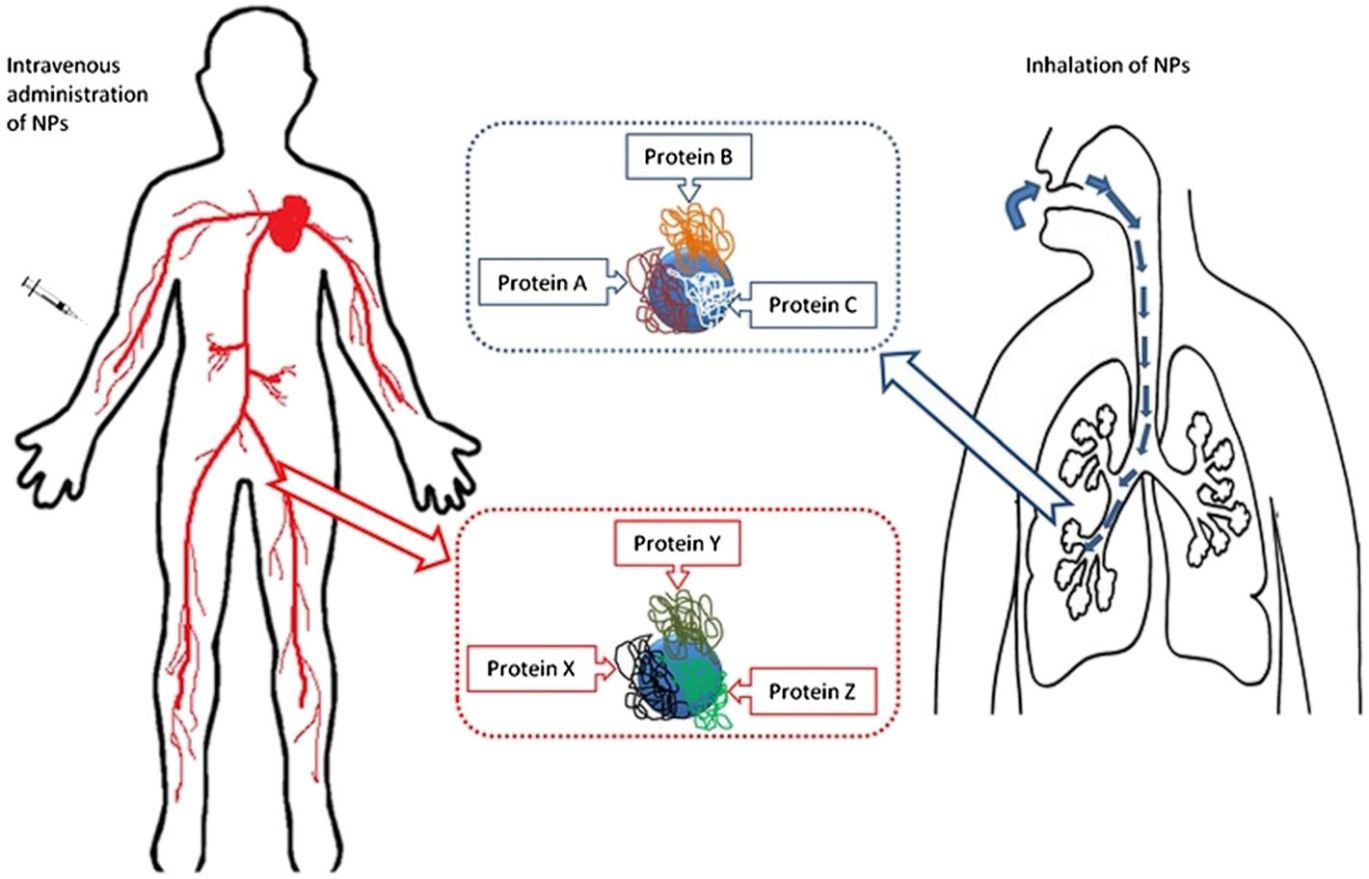
Schematic illustration of difference in the hard corona composition on nanoparticles depending on the route of administration; intravenous and inhalation. The proteins that adsorbs on nanoparticle may vary depending on its exposure to the different types of biological fluids in the human body

It was shown that assessment of protein corona composition in gradient plasma media is critical to understand what the cell “see” *in vivo* due to different pathways of NPs. When NPs enter the body, they will come into contact with a multitude of biological components in the bodily fluid before contacting the target cell. As they traverse in their path to reach the intended target site, the concentration and the type of proteins they encounter will also differ depending on the administrative route (*e.g.*, subcutaneous, intradermal, intramuscular, intravenous, intraosseous, intralumbar, and inhalation). Hence, the resulting corona formation on NPs would also be different [21, 24]. For example, the difference in the protein corona composition can be observed in the case of inhalation and intravenous route. In the case of NPs administered via the inhalation route, it will reach the lung cell barrier which contains different biological fluids, therefore accumulating totally different plasma proteins than the blood for NPs administered via the intravenous route as depicted in Fig. 2.

Based on their work, Ghavami and co-workers hypothesized that plasma protein gradient has great impact on the composition of protein corona and the biological fate of NPs *in vivo* [21]. They have employed two NPs, hydrophobic carboxylated polystyrene (PSOSO3) and hydrophilic silica (SiO2) particles, to probe the effect of the plasma concentration gradient. They used one-dimensional sodium dodecyl sulfate polyacrylamide gel electrophoresis (1D PAGE), liquid chromatography mass spectrometry (LC-MS/MS), dynamic light scattering (DLS), zeta potential, differential centrifugal sedimentation (DCS), and transmission electron microscopy (TEM) techniques to characterize NP-protein complexes. They have concluded that the composition and the quantity of proteins existing in the hard corona vary between the gradient plasma media and non-gradient plasma media. More specifically, the quantity of low molecular weight proteins (˂25 kDa) in the corona decreased compared to the amount that was forming in non-gradient plasma media [21].

##### Cell Observer

Another remarkable factor in determining the fate of NPs *in vivo* is “cell observer” (*i.e.*, cell types). The first contact point of cells with the surface of NP is the cell membrane which is distinct for each cell type due to the difference in surface proteins, sugars, and phospholipids. Large variation in cell membranes leads to different cellular uptake and toxicity mechanisms [29, 69]. The concept of “cell observer” should be taken into account in order to interpret the toxicity data as well as to determine the dose of NP per cell in therapeutic application of NPs. The impact of “cell observer” on the uptake and toxicity of superparamagnetic iron oxide NPs (SPIONs) were demonstrated by Laurent and co-workers [69]. SPIONs with different surface chemistries are interacted with a number of cell lines such as Capan-2, Panc-1, Hela, and Jurkat cells. It was reported that each cell line interacted with the NPs in a different way. For instance, while high level toxic effects could be observed on the brain–derived neuronal and glial cells and lung cells, same SPIONs caused moderate level toxic effects on the other cell types. In particular, both the uptake and toxicity of SPIONs are significantly dependent on the cell type.

##### Plasma Concentration Effect

**Fig. 3:**
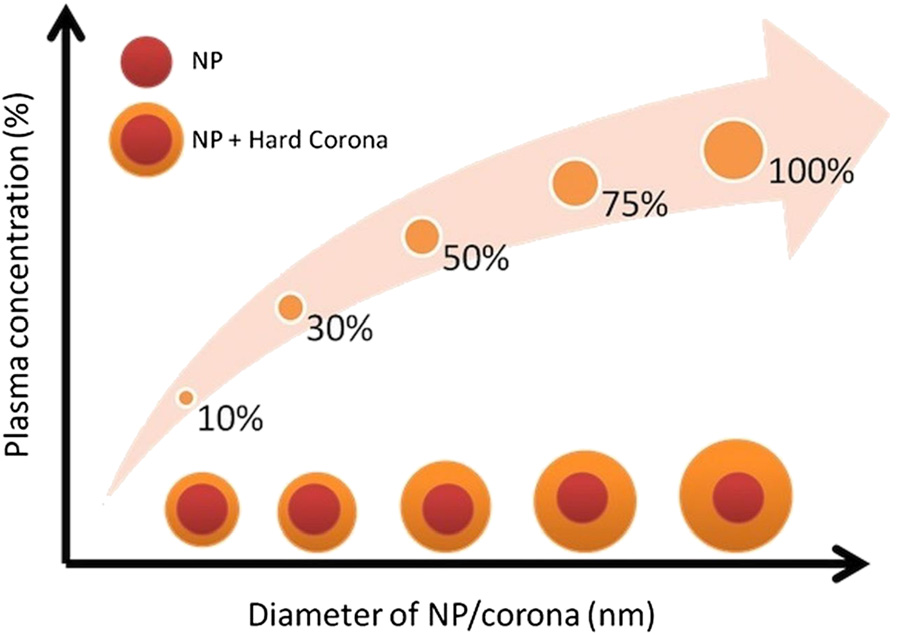
Graphical representation of plasma concentration effect on the thickness of hard corona formation on nanoparticles. The thickness of hard protein corona increases reciprocally as the concentration of plasma increases

The effect of plasma concentration on the composition of protein corona was investigated by Monopoli and co-workers [27]. They employed PSOSO3 NPs and hydrophilic silica (SiO2) NPs to study the protein adsorption and protein corona. They characterized NP-protein complex by DCS, DLS, and zeta potential, whereas composition of the hard corona was determined semi-quantitatively by using 1D PAGE and electrospray liquid chromatography mass spectrometry (LC MS/MS). They found that increasing the concentration of plasma increases the thickness of the hard protein corona as shown on Fig. 3. In addition, it was observed that the structure of NP-protein complexes in situ is roughly the same with the structure of those *in vitro* after separation from excess plasma. More specifically, the concept that the hard corona may evolve remarkably as a function of protein concentrations will have significant impact when studies conducted on *in vitro* cell culture conditions were used to extrapolate over to *in vivo* conditions.

##### Temperature

It is notable that the human body temperatures differ according to the parts of body [70], gender, and physical activities. When peripheral parts of the body are exposed to cold weather, body temperature decreases to 28 °C [71]. It has been shown that even the temperature of intracellular living cells is not homogeneous [71, 72]. Female’s body temperature is higher than the male body temperature, and it also changes with female’s hormonal cycle. Physical activities cause an increment of 2 °C and during sleep the temperature of body drops to a lower state. Moreover, fever causes the temperature of body to increase to 41 °C [73]. Hence, body temperature varies in the range of 35 to 39 °C. Mahmoudi and co-workers have studied the effect of temperature variation on the formation and composition of protein corona [74]. Fluorescently labeled, negatively charged polymer-coated FePt NPs were applied to incubate with human serum albumin (HSA) and fluorescence correlation spectroscopy (FCS) was used to quantify protein absorption at different concentrations and temperatures. Furthermore, Dextran-coated FeO_x_ NPs with different surface charges were incubated with FBS at different temperatures. It was concluded that the protein corona composition is influenced by variation in incubation temperature and has great impact on the cellular uptake as well.

The effect of plasmonic heat induction on the protein corona composition of gold nanorods was investigated by Mahmoudi and co-workers [75]. They have incubated cetyltrimethylammonium bromide (CTAB)-stabilized gold nanorods with FBS at different concentrations. Thereafter, protein corona composition before and after plasmonic heating induced by continuous laser irradiation were studied. UV-vis absorbance spectroscopy, transmission electron microscopy, ζ potential, and LC-MS/MS analysis were used to characterize the NP-protein complexes. They have revealed that the composition of hard protein corona was changed noticeably by applying both irradiation and thermal heating while there was no significant effect on the surface charge of protein corona. In contrast, differences in the composition of protein corona formed on gold nanorods were observed when different forms of heating were used as in the case of plasmonic heating (*i.e.*, photoinduced) and conventional thermal heating. They have concluded that alteration in the protein corona composition as a result of photoinduced local heating might affect the biological fate of NPs. Hence, these changes that will affect the final biological fate of plasmonic NPs should be taken into account for biological safety design and application of NPs for hyperthermia treatments.

##### Cell Membrane Composition

**Fig. 4:**
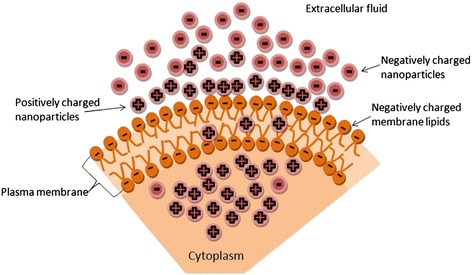
Scheme showing positively charged nanoparticles having a greater efficiency in cell membrane penetration and cellular internalization than negatively charged nanoparticles

In biological environments, surfaces of NPs are significantly modified by the adsorption of proteins leading to the creation of an interface between NPs and the cell membrane (CM) [25]. Therefore, to better understand the interactions that occur at the bio-nano interface, the effect of cell membrane should also be considered. For instance, it is well known that negatively charged CM causes positively charged NPs to have a greater efficiency in cell membrane penetration and cellular internalization than negatively charged NPs as depicted in Fig. 4. In other words, the negatively charged NPs have lower level of CM adsorption, which consequently decreases the probability of cellular uptake. Nevertheless, the cellular uptake rate of positively charged NPs can significantly disrupt the CM and as a result increases its toxicity [76, 77].

## Conclusions

In this review, we have highlighted the factors that affect protein corona formation on NPs based on the current knowledge and understanding of this unique phenomenon. Moreover, the section on the ignored factors has schemed through some of the issues and precautions that must be considered prior to the application of NPs in humans for medical treatment and diagnosis. Emphasis should be placed on more research to realize other hidden factors governing protein corona formation. With more information, the existing problems faced by researchers in this field could be rectified or solved and smarter solution are hoped to be found. Understanding protein corona formation and its biological consequences will be pivotal as the field of nanomedicine is set to dominate in the near future.
